# Obesity-related biomarkers underlie a shared genetic architecture between childhood body mass index and childhood asthma

**DOI:** 10.1038/s42003-022-04070-9

**Published:** 2022-10-17

**Authors:** Xikun Han, Zhaozhong Zhu, Qian Xiao, Jun Li, Xiumei Hong, Xiaobin Wang, Kohei Hasegawa, Carlos A. Camargo, Liming Liang

**Affiliations:** 1 Department of Epidemiology, Harvard T H Chan School of Public Health, Boston, Massachusetts USA; 2 Program in Genetic Epidemiology and Statistical Genetics, Harvard T H Chan School of Public Health, Boston, MA USA; 3 Department of Emergency Medicine, Massachusetts General Hospital, Harvard Medical School, Boston, MA USA; 4 Department of Biostatistics, Harvard T H Chan School of Public Health, Boston, MA USA; 5 Department of Nutrition, Harvard T H Chan School of Public Health, Boston, MA USA; 6 Center on the Early Life Origins of Disease, Department of Population, Family and Reproductive Health, Johns Hopkins Bloomberg School of Public Health, Baltimore, MD USA

**Keywords:** Genome-wide association studies, Asthma

## Abstract

Obesity and asthma are both common diseases with high population burden worldwide. Recent genetic association studies have shown that obesity is associated with asthma in adults. The relationship between childhood obesity and childhood asthma, and the underlying mechanisms linking obesity to asthma remain to be clarified. In the present study, leveraging large-scale genetic data from UK biobank and several other data sources, we investigated the shared genetic components between body mass index (BMI, *n* = 39620) in children and childhood asthma (*n*_case_ = 10524, *n*_control_ = 373393). We included GWAS summary statistics for nine obesity-related biomarkers to evaluate potential biological mediators underlying obesity and asthma. We found a genetic correlation (Rg = 0.10, *P* = 0.02) between childhood BMI and childhood asthma, whereas the genetic correlation between adult BMI (*n* = 371541) and childhood asthma was null (Rg = −0.03, *P* = 0.21). Genomic structural equation modeling analysis further provided evidence that the genetic effect of childhood BMI on childhood asthma (standardized effect size 0.17, *P* = 0.009) was not driven by the genetic component of adult BMI. Bayesian colocalization analysis identified a shared causal variant rs12436181 that was mapped to gene *AMN* using gene expression data in lung tissue. Mendelian randomization showed that the odds ratio of childhood asthma for one standard deviation higher of childhood BMI was 1.13 (95% confidence interval: 0.96–1.34). A systematic survey of obesity-related biomarkers showed that IL-6 and adiponectin are potential biological mediators linking obesity and asthma in children. This large-scale genetic study provides evidence that unique childhood obesity pathways could lead to childhood asthma. The findings shed light on childhood asthma pathogenic mechanisms and prevention.

## Abstract (web summary)

A comprehensive genetic analysis of the relationship between childhood obesity and childhood asthma identifies shared mechanisms and potential mediators linking the two pediatric disorders.

## Introduction

Asthma is one of the most common chronic respiratory diseases and affects more than 300 million people worldwide^1–3^. The prevalence of asthma has increased dramatically along with the epidemic of obesity in the recent decades, especially in developed western countries, causing the comorbidity obesity-asthma^4^. Asthma is a heterogeneous disease that often begins in childhood, but can also occur throughout life. Childhood-onset asthma typically has allergic comorbid conditions, such as atopic dermatitis and allergic rhinitis, whereas adult onset asthma is more likely to be a non-allergic phenotype^2,5^. Genetic studies have shown that childhood and adult onset asthma share a large proportion of genetic components, but are partly distinct^6,7^. Examining the genetic differences of coexistent diseases or traits (i.e., obesity) with childhood and adult onset asthma could provide insights into asthma pathogenic mechanisms, prevention, and treatment^8^.

Previous observational and genetic studies have shown an association between adult body mass index (BMI) and adult onset asthma^9–11^. A meta-analysis of 18 observational studies has reported that overweight or obesity are associated with a higher risk of wheezing and asthma in children^12^. However, the traditional observational study design is prone to confounding and reverse association. Previous genetic studies for the association between BMI and asthma did not match adult and childhood BMI with adult and childhood asthma. An age-concordant (childhood and adult) genetic effect of BMI and asthma remains to be clarified^13–19^. For example, Au Yeung et al.^14^ evaluated the association between childhood BMI and asthma (all asthma cases in UK Biobank), where the study did not distinguish adult and childhood asthma. With the different genetic components of adult and childhood asthma, the effect of childhood BMI on childhood asthma was unexplored in their study. A summary of previous relevant studies and their limitations were summarized in Supplementary Table 1. In the current study, we investigate the role of childhood BMI on childhood-onset asthma, their shared genetic components, and the underlying biological mechanisms.

**Fig. 1:**
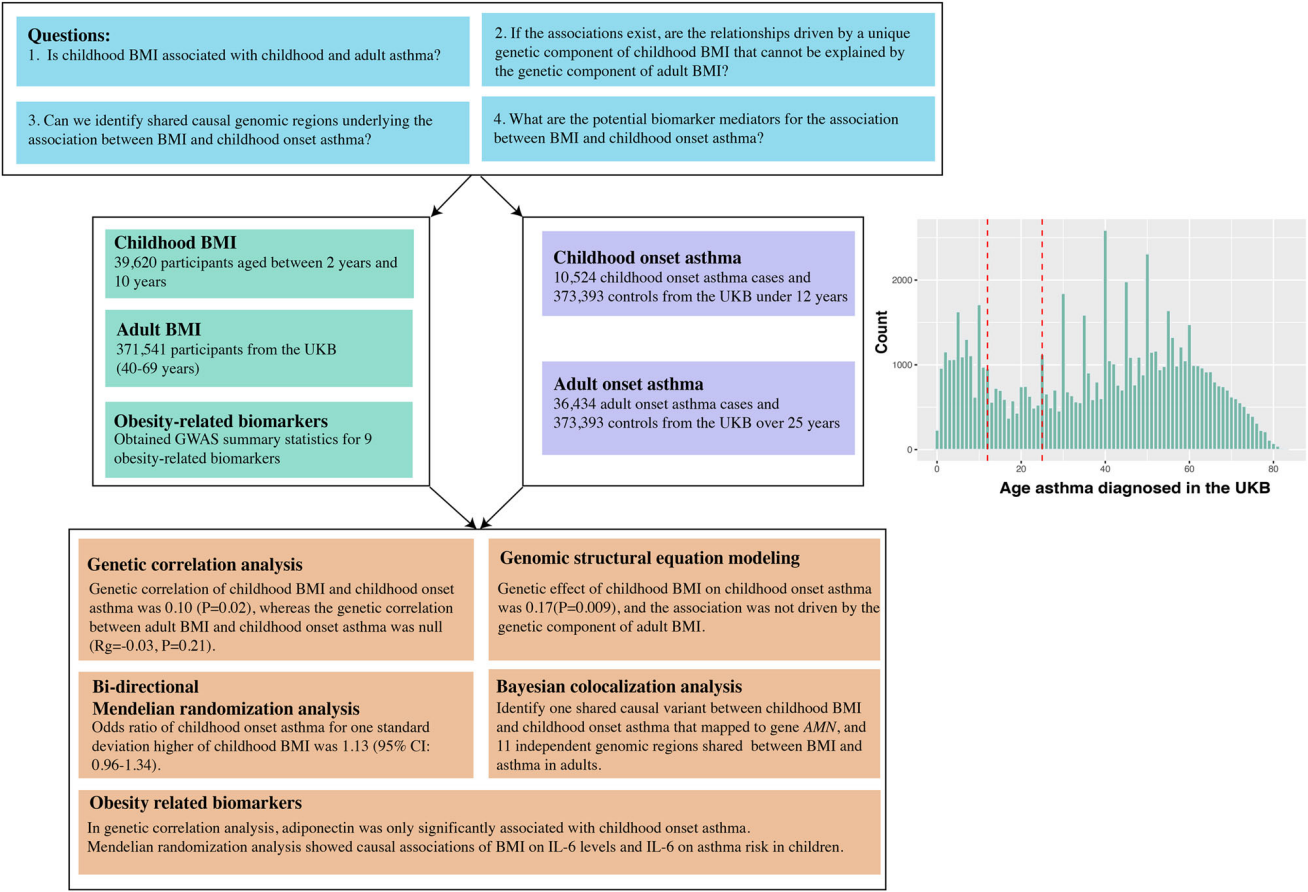
Study summary flowchart.

Here, leveraging large-scale genetic data and obesity-related biomarkers, we explore the shared genetic components between BMI and childhood-onset asthma. Specifically, we aim to address four questions (Fig. 1). First, is childhood BMI associated with childhood- and adult-onset asthma? Second, if the associations exist, are the relationships driven by a unique genetic component of childhood BMI that cannot be explained by the genetic component of adult BMI? Third, can we identify shared causal genomic regions underlying the association between BMI and childhood-onset asthma? Finally, what are the potential biomarker mediators for the association between BMI and childhood-onset asthma? Solving these questions would provide evidence for asthma prevention in children, bring insights into the shared genes for BMI and asthma, and reveal potential biological mediators linking obesity to asthma.

## Results

### Genome-wide genetic correlation between BMI and asthma

**Fig. 2:**
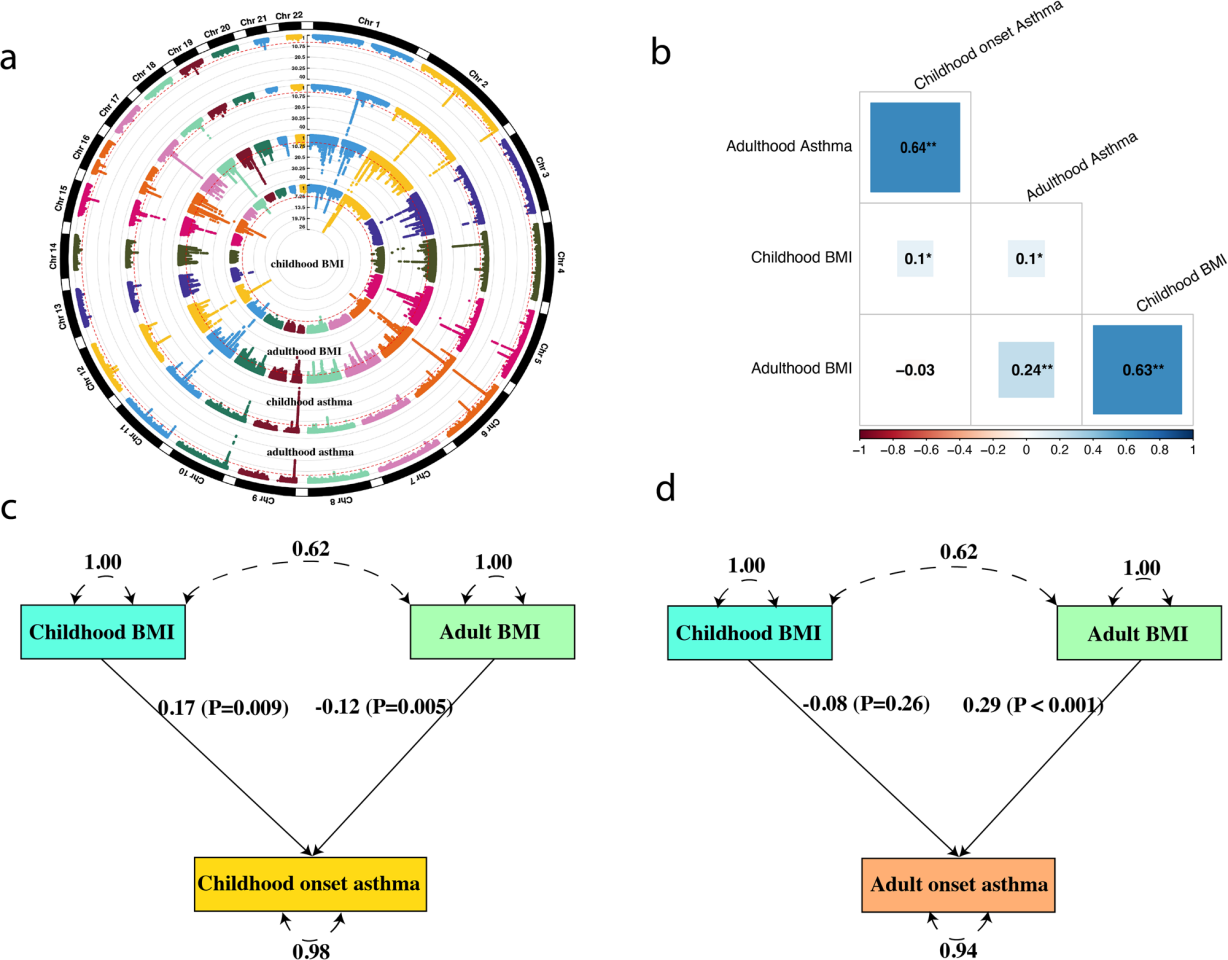
Genetic association between body mass index and childhood- and adult-onset asthma. **a** The circle Manhattan plot shows *P*-values of genome-wide association studies (GWAS) for body mass index (BMI) and childhood- and adult-onset asthma. From inner to outer circle: childhood BMI, adult BMI, childhood-onset asthma, and adult onset asthma. The circle Manhattan plot is customized to truncate the *P*-values at 1 × 10^−40^. **b** The genetic correlation coefficients for each pair of traits. The nominal significance level of genetic correlation (*P* < 0.05) is marked by one asterisk, and *P* < 0.01 is marked by two asterisks. **c**, **d** Genetic multivariable regression in genomic structural equation modeling evaluates the association between BMI and childhood- and adult-onset asthma. **c** The association between childhood BMI and childhood-onset asthma adjusting for the genetic effects of adult BMI. **d** The association between childhood BMI and adult onset asthma adjusting for the genetic effects of adult BMI.

The genome-wide association study (GWAS) of BMI and asthma in children and adults is presented in Fig. 2a. We evaluated the genetic correlation between BMI and asthma (Fig. 2b and Supplementary Table 2). For childhood BMI, the genetic correlation with childhood and adult onset asthma was 0.10 (*P* = 0.02) and 0.10 (*P* = 0.03), respectively. In contrast, the genetic correlation between adult BMI and childhood-onset asthma was null (Rg = −0.03, *P* = 0.21). Adult BMI was associated with adult onset asthma (Rg = 0.24, *P* = 1.70 × 10^−18^), consistent with previous studies^10,11^. Finally, the genetic correlations between childhood and adult for BMI or asthma were high (i.e., childhood and adult BMI Rg = 0.63, *P* = 2.46 × 10^−107^; childhood and adult onset asthma Rg = 0.64, *P* = 4.52 × 10^−43^), implying that the genetic architectures in different life stages largely overlap, but also have partly distinct genetic components.

### Genetic multivariable regression in genomic structural equation modeling (genomic SEM)

With the high genetic correlation between childhood BMI and adult BMI, it remained unclear whether the associations of childhood BMI with childhood-onset asthma and adult onset asthma were driven by the genetic component specific to adult BMI. We performed a genetic multivariable regression analysis in the genomic SEM framework to regress childhood-onset asthma on childhood BMI, allowing a partial genetic correlation between childhood BMI and adult BMI, and the genetic contribution from adult BMI to childhood-onset asthma (Fig. 2c). The genetic effect of childhood BMI on childhood-onset asthma was comparable to the genetic correlation estimates (standardized effect size 0.17, SE = 0.06, *P*-value = 0.009), showing that the association between childhood BMI and childhood-onset asthma was not primarily driven by the genetic components of adult BMI. In the multivariable regression analysis of genomic SEM, we also observed a negative effect of adult BMI on childhood-onset asthma (Fig. 2c). Given the null association in the bivariate genetic correlation between adult BMI and childhood-onset asthma (Fig. 2b), the negative effect in the multivariable regression genomic SEM analysis was likely induced by the high genetic correlation between adult BMI and childhood BMI.

Subsequently, we fitted a genetic multivariable regression model for adult onset asthma to simultaneously regress on childhood BMI and adult BMI (Fig. 2d). The conditional standardized association between childhood BMI and adult onset asthma was not significant (BETA = −0.08, SE = 0.07, *P* = 0.26). In contrast, the association between adult BMI and adult onset asthma remained robust (standardized effect size 0.29, SE = 0.05, *P* = 6.14 × 10^−9^). The results showed that after accounting for the high genetic correlation between childhood BMI and adult BMI, the direct effect of childhood BMI on adult onset asthma approached zero, indicating that the genetic correlation observed between childhood BMI and adult onset asthma was mainly driven by the genetic component related to adult BMI instead of the genetic component unique to childhood BMI.

### Mendelian randomization analysis to evaluate the putative causal association

**Fig. 3:**
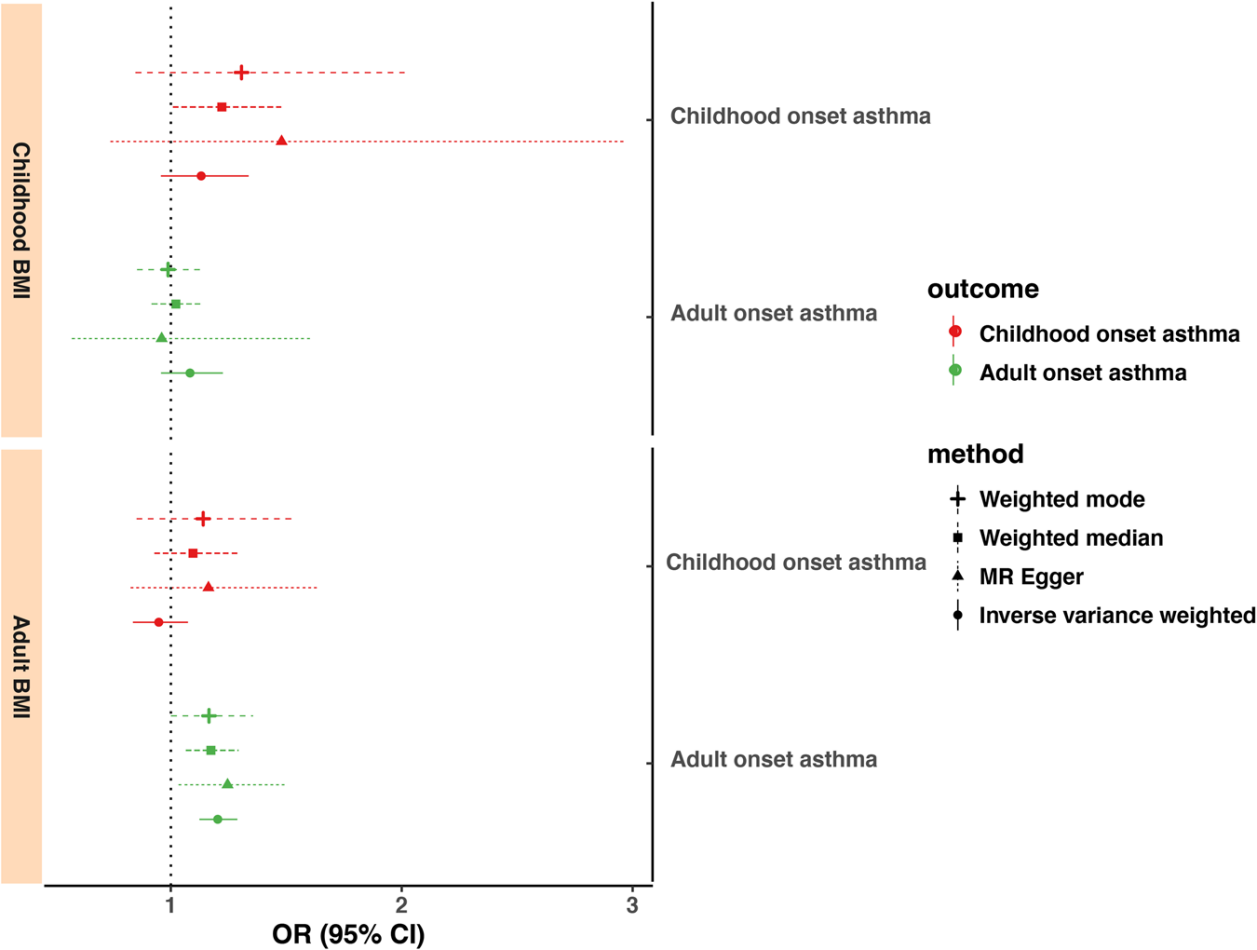
Mendelian randomization estimates of the association between body mass index and childhood- and adult-onset asthma. The *x*-axis is the odds ratio (OR) of the effects for per standard deviation increase in BMI (exposure) on asthma (outcome) in children and adults. The vertical dashed line is the reference at OR = 1. Different Mendelian randomization methods are displayed in different line types.

In the Mendelian randomization (MR) analysis, we first replicated the effect of adult BMI on adult onset asthma (for per standard deviation unit increase of BMI, MR-IVW odds ratio [OR] = 1.20, 95% confidence interval [CI]: 1.12–1.29, *P* = 9.45 × 10^−8^, Fig. 3), consistent with previous studies^10,11^. We observed that the OR of childhood-onset asthma for one standard deviation higher level of childhood BMI was 1.13 (from MR-IVW method, 95% CI: 0.96–1.34, *P* = 0.15; from MR-weighted median method, OR = 1.22, 95% CI: 1.00–1.48, *P* = 0.04) with a substantially wider confidence interval, reflecting the relatively smaller sample size and less genetic variants for childhood BMI compared with adult BMI. For childhood BMI, the F statistics and the proportion of variance explained *R*^2^ were 65 and 2.7%, respectively. The magnitudes of effect sizes from different MR methods were broadly consistent (Fig. 3). There was no evidence of directional pleiotropy effects from the MR-Egger intercept or heterogeneity from the MR-PRESSO outlier test. No robust associations were observed between childhood BMI and adult onset asthma, adult BMI and childhood-onset asthma, or in the reverse-directional MR analyses (Supplementary Fig. 1). Sensitivity analysis using the GIANT consortium adult BMI GWAS showed similar results.

### Shared causal variants between obesity and asthma

**Fig. 4:**
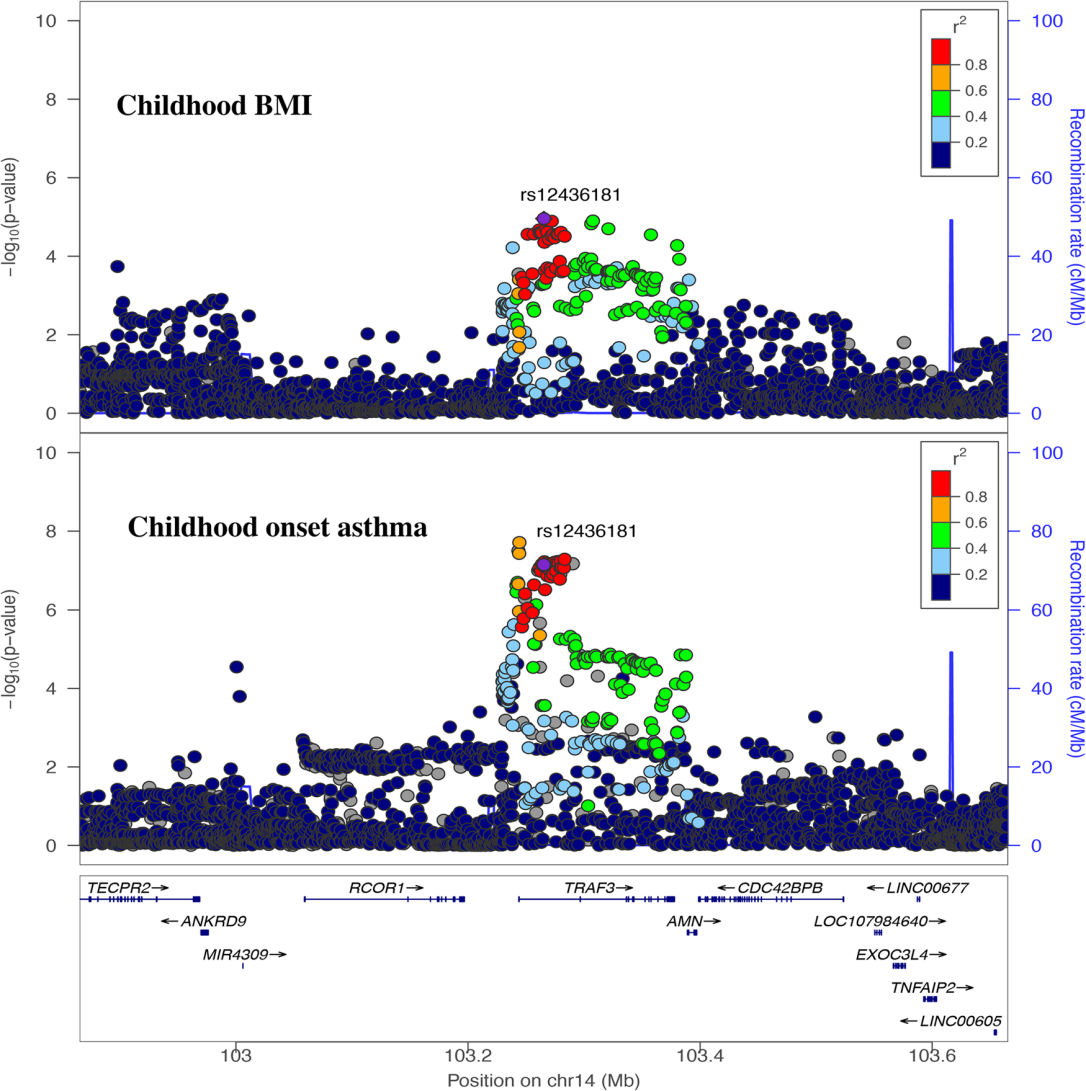
Bayesian colocalization analysis identifies a shared causal variant between childhood BMI and childhood-onset asthma.

In the Bayesian colocalization analysis, we identified one genomic region with a higher posterior probability (PP4 = 0.80) of shared causal variant between childhood BMI and childhood-onset asthma (Fig. 4). The SNP rs12436181 (near genes *TRAF3, AMN*) was associated with childhood-onset asthma, eczema, body size at age 10 years, and weight in the Open Targets Genetics^20^. In the Genotype-Tissue Expression (GTEx) database^21^, rs12436181 is associated with the gene expression of *AMN* in lung tissue (effect size 0.11, *P* = 7.9 × 10^−5^). Transmembrane protein amnionless (AMN) is essential for vitamin B_12_ uptake, which has potential effects on asthma^22,23^. We further evaluated the shared genomic regions between BMI and adult onset asthma and identified 11 shared genomic regions (Supplementary Fig. 2).

### Genetic association between obesity-related biomarkers and asthma

**Fig. 5:**
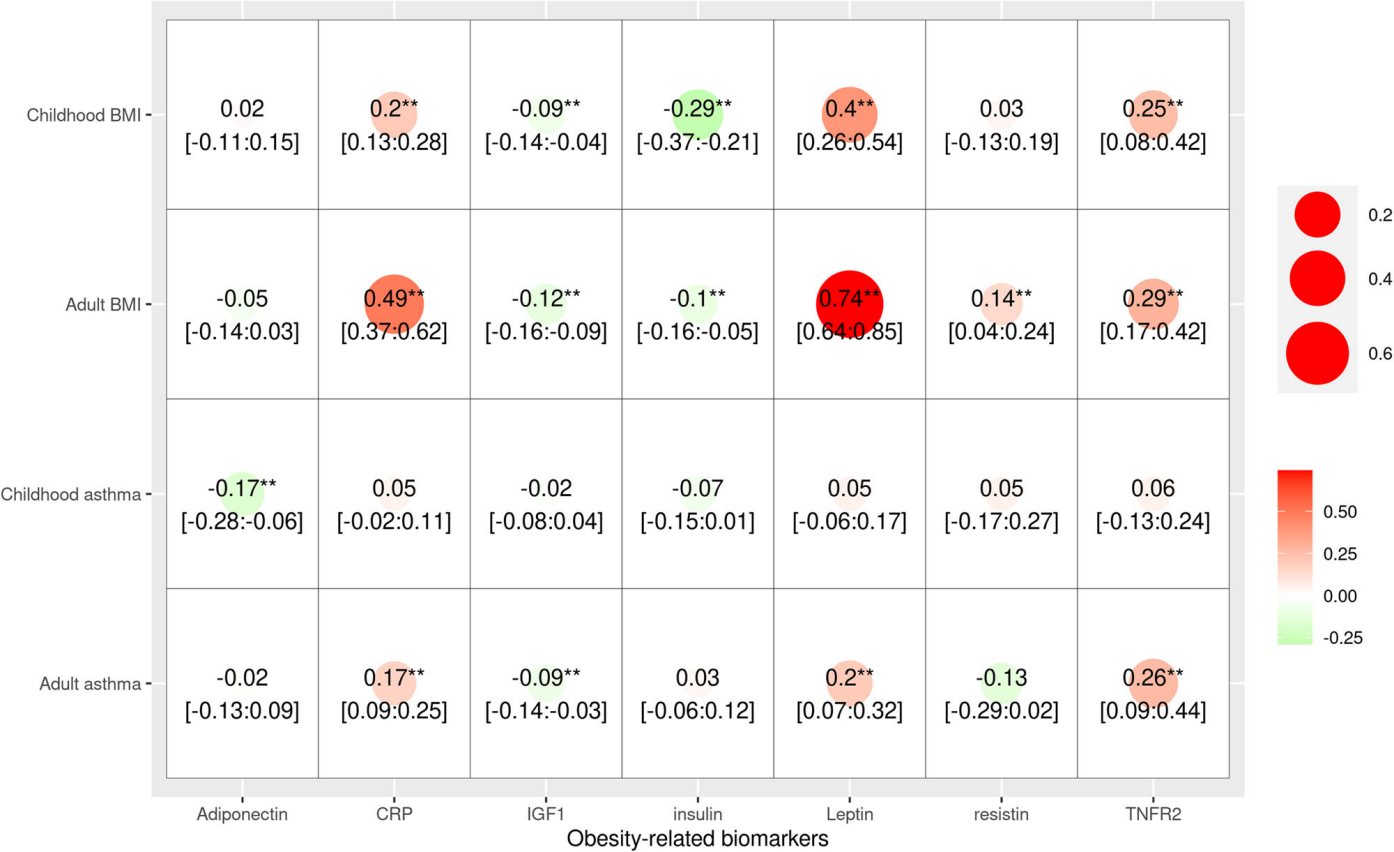
Genome-wide genetic correlation between obesity-related biomarkers and childhood- and adult-onset asthma. The direction of genetic correlation is presented in different colors, and the magnitude of genetic correlation is shown in different point sizes. The genetic correlation coefficients and the 95% confidence intervals (CI) are shown in each cell. The nominal significance level of genetic correlation (*P* < 0.05) is marked by one asterisk, and *P* < 0.01 is marked by two asterisks. The calculation of genetic correlation for IL-6 and TNFR1 failed due to computing issues.

**Fig. 6:**
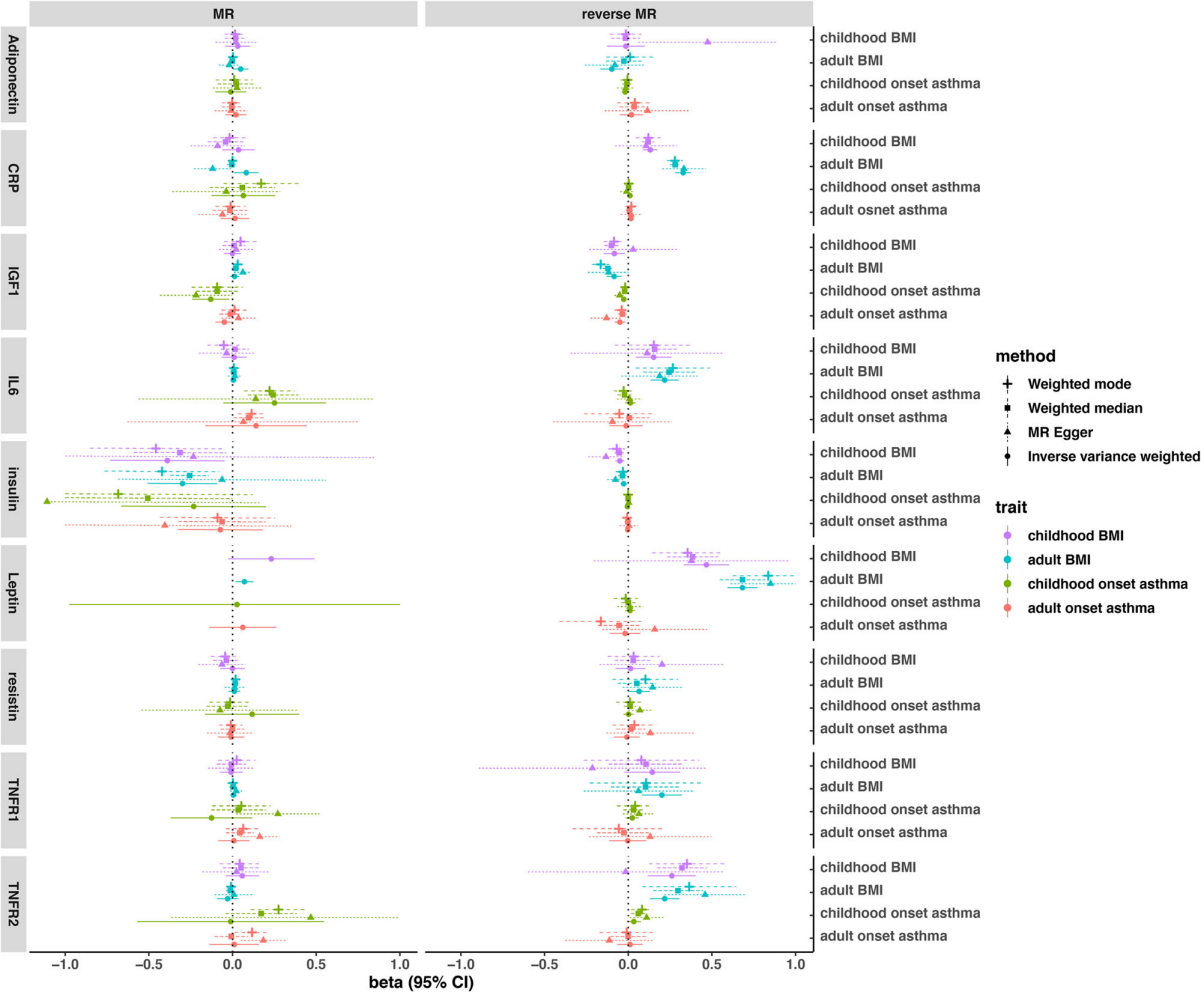
Bi-directional Mendelian randomization estimates of the association between asthma, BMI, and obesity-related biomarkers in children and adults. The MR panel shows the effects of obesity-related biomarkers (exposures) on risk of BMI and asthma (outcomes) in children and adults. The reverse MR panel shows the effects of BMI and asthma on obesity-related biomarkers (reverse-directional MR analysis). Different Mendelian randomization methods are displayed in different line types. For the binary exposures (childhood and adult asthma in the reverse MR analysis), MR estimates here are only used to evaluate evidence for causality and the consistency of direction of effect. The interpretation of effect sizes for binary exposures needs more caution.

To further investigate the mechanism between age-specific obesity and asthma, we systematically evaluated the association of obesity-related biomarkers with asthma and BMI in children and adults. In the genetic correlation analysis, we found that adiponectin was only significantly associated with childhood-onset asthma, whereas CRP, IGF-1, leptin, and TNFR2 were only significantly associated with adult onset asthma (Fig. 5). In the MR analysis, we identified putative causal associations of BMI on IL-6 levels and IL-6 on asthma risk in children after multiple testing, whereas the association of IL-6 with adult onset asthma was nominally significant (Fig. 6). In the reverse MR analysis, we also observed effects of BMI (both childhood and adult) on obesity-related biomarkers, such as CRP, IGF1, insulin, leptin, and TNFR2 (Fig. 6). These results were essentially the same after adjusting for sample overlap. We also replicated the association of IL-6 with asthma and wheezing in the Boston Birth Cohort (BBC). For one unit increase of IL6 (after log transformation), the odds ratio of both asthma and wheezing was 1.28 (95% CI: 1.03–1.59, *P* = 0.026, Supplementary Fig. 3).

## Discussion

In this study, we have shown that the shared genetic component between childhood BMI and childhood-onset asthma is not driven by the genetic component of adult BMI. We identified one shared causal genomic region between BMI and asthma in childhood that mapped to gene *AMN*. To dissect the underlying biological mediators between obesity and asthma, we systematically evaluate obesity-related biomarkers and show IL-6 linking childhood obesity to childhood-onset asthma. The findings support the important role of obesity in childhood-onset asthma and provide insights into childhood-onset asthma pathogenic mechanisms and prevention.

Our study presented an important contribution to our understanding of the role of childhood BMI on childhood-onset asthma, their shared genetic components, and the underlying biological mechanisms. Our study corroborated results from previous observational and genetic studies that BMI was associated with asthma in *adults*^9–11^. For instance, in a previous MR analysis, Zhu et al.^10^ reported that adult BMI was associated with late-onset asthma (OR = 1.21, *P* = 6.3 × 10^−7^). Another MR study showed that the OR of BMI on asthma risk (a general definition included asthma in children and adults) was 1.18 (*P* = 2 × 10^−8^)^11^. A recent MR study has suggested an association of childhood adiposity with asthma (asthma cases with heterogeneous onset stages), however, asthma risk in children was not examined^14^. Richardson et al.^18^ have shown that childhood body size increased asthma risk (asthma cases with heterogeneous onset stages), and their multivariable MR analysis indicated that childhood body size indirectly influences asthma risk via adult body size. The genetic evidence for the association between obesity and childhood-onset asthma remains unclear. Previous studies have shown that the genetic architecture of obesity and asthma in children and adults overlap, but is also partly distinct^6,24,25^. Between 5 years and 7 years of age, BMI reaches a minimum point in children (adiposity rebound), and then starts to rise during adolescence^26^. For asthma, childhood-onset is more likely to have allergic comorbid conditions, while adult onset asthma is more likely to be a non-allergic phenotype^1^. In the current genetic analysis, we found that the association between childhood BMI and childhood-onset asthma was weaker than the association in adults (MR-IVW OR 1.13 versus 1.20). In the genomic structural equation modeling, we showed that the association of childhood BMI with childhood-onset asthma was not driven by the genetic component of adult BMI. The shared genetic components between childhood BMI and childhood asthma remain unclear. Previous studies^16,17^ have reported BMI associated genes in asthmatic children, in our current study, we performed a formal colocalization analysis using GWAS summary statistics, and identified a shared causal genomic region between BMI and asthma in childhood that mapped to gene AMN.

We have shown IL-6 as a potential mediator linking obesity and asthma. Previous genetic studies have reported that IL-6 increased risk of asthma^27,28^. One major source of IL-6 is from M1 macrophages in adipose tissue, and IL-6 is potential to be the underlying biological mediator to link obesity and asthma by IL-6 trans-signaling pathway and airway inflammation^29,30^. We observed a negative genetic correlation between adiponectin and childhood-onset asthma, which is consistent with the fact that the circulating levels of adiponectin are reduced in obesity^31^. Adiponectin has anti-inflammatory effects, such as inhibiting airway hyperreactivity, eosinophil influx, and type 2 cytokines, in response to allergen^31^. Altogether, our study suggested that the underlying mechanisms for the association between childhood BMI and childhood-onset asthma might involve IL-6 and adiponectin.

The findings from this study have important public health and clinical implications. First, from the point view of prevention, one critical question is whether we can incorporate early life weight management into current childhood-onset asthma prevention strategies. Second, obesity and its related biomarkers could shed light on asthma subtypes. Further studies are warranted to investigate asthma treatment effectiveness in childhood-onset asthma patients with obesity, early lifestyle weight-loss intervention strategies, and molecular therapeutic targets, such as adiponectin and the IL-6 trans-signaling pathway.

A key strength of the present study is that we used large-scale genetic data to evaluate the shared genetic component between childhood obesity and childhood-onset asthma. The identified genetic variants and biomarkers shared between obesity and asthma have potential biological and therapeutic utilities to extend the understanding of asthma prevention and treatment in children. In addition, compared with traditional observational analysis, an MR study is less likely to be affected by confounding or reverse causation. At the same time, there are several limitations of this study. First, the genetic correlation and MR analysis are unable to evaluate a non-linear relationship between childhood BMI and childhood-onset asthma. While BMI is relatively stable in adults, the obesity rebound in children could make the linear assumption more complex. Second, the sample size of childhood BMI GWAS used in this study is much smaller than adult BMI. Third, we only included genetic data from European participants, the generalizability to non-European populations remains unclear. Forth, asthma severity and different asthma endotypes (eg. allergic vs non-allergic asthma; eosinophilic vs non-eosinophilic asthma) are not accounted for in this study, further subtype GWAS analysis would provide further evidence. For example, it is likely that obesity is correlated with non-allergic and type 2 low asthma^32^. Fifth, the genetic effect on obesity-related biomarker data were derived from adult populations and the umbilical IL-6 concentrations in BBC might not represent the level during early childhood, age-specific biomarker data will provide a full view of the effects of childhood biomarkers on childhood asthma. Finally, the asthma cases ascertained from the UKB include self-reported cases. However, a previous study has shown that self-reported data can improve power and have a high genetic concordance with hospital diagnosis^33^.

In conclusion, this study contributes genetic evidence that obesity increases the risk of asthma in children. The association between childhood BMI and childhood-onset asthma is not driven by the genetic component of adult BMI. IL-6 and adiponectin are potential biological mediators for the association. The findings shed light on childhood asthma pathogenic mechanisms, prevention, and treatment.

## Methods

### Body mass index

BMI was used as the main measure of obesity. For childhood BMI, we downloaded the publicly available genome-wide association study (GWAS) summary statistics for 8,227,807 single-nucleotide polymorphisms (SNPs) from a discovery meta-analysis of 39,620 participants aged between 2 years and 10 years^25^. In the GWAS, the childhood BMI was transformed into the scale of standardized deviation score after adjusting for age and sex. Genotype data were imputed to the 1000 Genomes Project or the Haplotype Reference Consortium. The SNPs were filtered with minor allele frequency more than 1% and imputation quality score more than 0.6.

For adult BMI, we used data from the UK Biobank (UKB), a large-scale prospective cohort study with detailed phenotypic and genetic data on half a million participants aged between 40–69 years across the United Kingdom^34^. We only included European participants based on a K-means clustering algorithm of genetic components and self-reported ancestry information (UKB data field 22009 and 21000)^35^. To avoid potential bias, we removed all asthma cases in the adult BMI GWAS (see section, Childhood- and adult-onset asthma cases, below). In total, 371,541 participants from the UKB data field 21001 were selected in the adult BMI GWAS. As described in a previous study^36^, the sample overlap only in controls can avoid potential bias in Mendelian randomization (MR) analysis. A rank-based inverse normal transformation was applied to BMI before the GWAS analysis to interpret genetic effect sizes in a standard deviation unit^37^. In our sensitivity analysis, we also used adult BMI GWAS summary statistics from the Genetic Investigation of ANthropometric Traits (GIANT) consortium (2015 release without UK Biobank participants) with approximately 2 million SNPs^38^.

The UK Biobank has been reviewed and approved by local research ethics boards. All participants provided informed written consent, and study procedures were performed in accordance with the World Medical Association Declaration of Helsinki ethical principles for medical research.

### Obesity-related biomarkers

BMI is an imperfect measure of obesity and might not reflect the underlying biological mechanisms for risk of disease outcomes^39^. To investigate the potential biological mediators for the association between obesity and asthma, we obtained available GWAS summary statistics (described in Supplementary Note 1, Supplementary Table 3) for obesity-related biomarkers in two major pathways: insulin/insulin-like growth factor (IGF) axis and chronic low-grade inflammation, including adiponectin, C-reactive protein (CRP), IGF-1, interleukin 6 (IL-6), insulin, leptin, resistin, and tumor necrosis factor receptors (TNFR1, TNFR2)^39^.

### Childhood- and adult-onset asthma cases

We defined childhood- and adult-onset asthma cases using individual level data from UKB and conducted GWAS analysis. The GWAS analysis allowed us to avoid sample overlap with adult BMI in UKB and to perform various sensitivity analyses, while publicly available GWAS summary statistics for asthma did not meet the requirements. Childhood- and adult-onset asthma cases were ascertained from the UKB where health-related outcomes were collected through hospital inpatient records, primary care data, cancer/death registry records, and self-reports of doctor diagnoses based on touchscreen questionnaires^34,40^. A detailed first occurrence of health outcome coding classifications was developed (https://biobank.ctsu.ox.ac.uk/crystal/ukb/docs/first_occurrences_outcomes.pdf) to harmonize different coding systems (International Classification of Diseases [ICD] codes and Read codes). The first occurrences of asthma via UKB data fields 131494 and 131495 were used as the primary source to ascertain asthma cases. When the data fields were unavailable, we used another algorithm-defined date of asthma (data field 42014) developed by the UKB outcome adjudication group. We excluded 585 participants with >10 years difference of age at diagnosis between the two data fields 131494 and 42014. In this study, childhood- and adult-onset asthma were defined as age at diagnosis under 12 years and over 25 years, respectively^7,41^. Young adult-onset asthma (age of onset 12–25 years) was excluded in this study due to its high heterogeneity^6,41^. We noted that the age of first occurrence of asthma could be as early as less than 1 year, therefore, we further restricted the age of first occurrence between 5 and 11 years for childhood-onset asthma cases in our sensitivity analysis. We excluded participants (*n* = 27,434) with chronic obstructive pulmonary disease, emphysema, or chronic bronchitis from this study^6,7,41,42^. In total, we included 10,524 childhood-onset asthma cases, 36,434 adult onset asthma cases, and 373,393 shared controls after restricting to participants of European ancestry.

The UK Biobank was approved by the National Research Ethics Service Committee North West–Haydock, in accordance with the Declaration of Helsinki.

#### Boston birth cohort

To further investigate the association between IL6 and asthma, we included the Boston Birth Cohort (BBC), a prospective birth cohort study initiated in 1998 under rolling enrollment at Boston Medical Center. The detailed information of the BBC was described in previous studies^43–45^. In the current study, we included 394 children with both IL-6 and asthma information. In the BBC, umbilical cord blood was collected at delivery to measure IL-6, which was quantified by immunoassay using flowmetric Luminex xMAP technology (Luminex Corp, Austin, TX)^44^. Asthma and wheezing were defined using physician diagnosis code (International Classification of Diseases, Ninth Revision) in children 2 years and older^45^.

### Statistics and reproducibility

#### Genome-wide association analysis

Genome-wide association analysis was performed in the REGENIE software (v2.0.2), a computationally efficient whole-genome regression for both quantitative and binary traits^46^. For adult BMI GWAS in the UKB, linear regression tests were conducted, whereas for the binary outcome asthma GWAS, approximate Firth logistic regression tests were performed. For all GWAS, we adjusted for sex, age at recruitment, age squared, genetic batch effect, and the first 20 genetic components. Genetic variants with minor allele frequency less than 0.01 and imputation quality score less than 0.6 were excluded from the association tests.

#### Genetic correlation

We used LD Score regression (LDSC, v1.0.1) to evaluate the genetic correlation between BMI and childhood- and adult-onset asthma, respectively^47,48^. The BMI and asthma GWAS summary statistics were filtered to 1.2 million HapMap3 SNPs without the major histocompatibility complex region. The default parameters and LD scores from European participants were used^47,48^.

#### Genomic structural equation modeling

Genomic structural equation modeling (genomic SEM) is a multivariate method to model genetic covariance based on GWAS summary statistics regardless of sample overlap^49^. In this study, a genetic multivariable regression analysis was conducted in the genomic SEM framework (R package GenomicSEM, v0.0.5) to disentangle whether the effect of childhood BMI on childhood- and adult-onset asthma was partly explained by the genetic component of adult BMI. Briefly, a built-in LDSC function was used to compute the genetic covariance matrix between a series of input traits, allowing an arbitrary degree of sample overlap. A customized multivariate model can be fitted based on the genetic covariance matrix to explore direct genetic effects of childhood BMI on childhood- and adult-onset asthma adjusting for genetic effects of adult BMI.

#### Bi-directional Mendelian randomization analysis

A two-sample MR analysis was performed to investigate the putative causal effect between childhood BMI and childhood-onset asthma^50,51^. For BMI (as the exposure), we selected genome-wide significant independent SNPs (*P* < 5 × 10^−8^, *r*^2^ < 0.001) as genetic instrumental variables in the MR analysis. The F statistics and proportion of variance explained (*R*^2^) were calculated to evaluate the strength of genetic instruments^52^. The inverse-variance weighted (MR-IVW) method was used as the initial analysis where the genetic effects of genetic instruments on childhood-onset asthma (outcome) were regressed against the genetic effects on childhood BMI (exposure), based on a weighted linear regression model with a zero intercept term^50^. We also conducted a series of sensitivity MR analyses to assess the robustness of IVW results that allow violations of MR assumptions, including weighted mode, weighted median, and MR-Egger^53–55^. The intercept term from the MR Egger regression was used to evaluate potential directional horizontal pleiotropy (i.e., intercept close to zero and *P*-value > 0.05)^54^. We also performed the MR pleiotropy residual sum and outlier (MR-PRESSO) approach to identify potential bias from outlier variants (MR-PRESSO outlier test) and assess the overall heterogeneity of the MR estimates (MR-PRESSO distortion test)^55^. Reverse-directional MR analyses were used to evaluate the effects of asthma on risk of obesity or obesity-related biomarkers^56^. To adjust for multiple testing, a false discovery rate (FDR) method was used to prioritize obesity-related biomarkers, accounting for the number of traits (9 biomarkers, childhood/adult BMI/asthma), MR methods (4 MR methods), and directions of MR analysis.

In the MR analysis for adult BMI and asthma, the sample overlap of BMI only in controls can avoid potential bias due to sample overlap^36^. For obesity-related biomarkers, GWAS summary statistics for IGF-1 and CRP were from the UKB, where sample overlap with our BMI and asthma GWAS can bias MR estimates. A cross-trait LD-score regression method was used to approximate an arbitrary degree of sample overlap and to correct the bias^57^.

#### Shared genomic regions between obesity and asthma

We conducted a Bayesian colocalization analysis using COLOC (R package, version 5.1.0) to investigate the shared causal variants between childhood BMI and childhood-onset asthma^58^. To identify shared variants, we selected independent index SNPs for childhood BMI and childhood asthma based on SNP *P*-value < 1 × 10^−5^ and *R*^2^ < 0.01. The tested genomic region included all SNPs within a window of 1 Mb each side from the index SNP. In the colocalization analysis, the posterior probabilities were calculated for five hypotheses: H0, no association with either trait; H1 and H2, association with trait 1 or trait 2; H3, association with both traits but two independent causal variants; and H4, association with both traits with a shared causal variant. A posterior probability of H4 (PP4) higher than the other four hypotheses supported a shared causal variant affecting both traits.

### Reporting summary

Further information on research design is available in the Nature Research Reporting Summary linked to this article.

## Supplementary information

Supplementary Information (new)

Description of Additional Supplementary Files

Supplementary Data 1 (new)

reporting summary

## Data Availability

UK Biobank data are available through the UK Biobank Access Management System https://www.ukbiobank.ac.uk/. Data for Figs. 3 and 6 were provided in Supplementary Data 1.

## Code availability

The following software packages were used for data analyses: LOCUSZOOM: http://locuszoom.sph.umich.edu/; LD score regression software: https://github.com/bulik/ldsc; METAL software: http://csg.sph.umich.edu/abecasis/Metal/; PLINK software: http://www.cog-genomics.org/plink2; R: https://cran.r-project.org/; REGENIE software: https://rgcgithub.github.io/regenie/overview/.
