# Supplementary material for: Obesity-related biomarkers underlie a shared genetic architecture between childhood body mass index and childhood asthma
Source: Commun Biol. 2022 Oct 17;5:1098. doi: 10.1038/s42003-022-04070-9 (PMC9576683; doi:10.1038/s42003-022-04070-9)
Supplement: Supplementary file 1 — Supplementary Information (new) [file 42003_2022_4070_MOESM1_ESM.pdf]

Supplementary Information

Obesity-related biomarkers underlie a shared genetic architecture between childhood body mass index and childhood onset asthma

Xikun Han,[1,2] Zhaozhong Zhu,[3] Qian Xiao,[4] Jun Li,[5] Xiumei Hong,[6] Xiaobin Wang,[6] Kohei Hasegawa,[3] Carlos A Camargo,[1,3] Liming Liang[1,2,4]

Affiliations:

1. Department of Epidemiology, Harvard T H Chan School of Public Health, Boston, Massachusetts, USA.
2. Program in Genetic Epidemiology and Statistical Genetics, Harvard T H Chan School of Public Health, Boston, Massachusetts, USA.
3. Department of Emergency Medicine, Massachusetts General Hospital, Harvard Medical School, Boston, Massachusetts, USA.
4. Department of Biostatistics, Harvard T H Chan School of Public Health, Boston, Massachusetts, USA.
5. Department of Nutrition, Harvard T H Chan School of Public Health, Boston, Massachusetts, USA.
6. Center on the Early Life Origins of Disease, Department of Population, Family and Reproductive Health, Johns Hopkins Bloomberg School of Public Health, Baltimore, MD, USA.

Supplementary Figure 1. Reverse directional Mendelian randomization estimates of the association between asthma and body mass index in children and adults

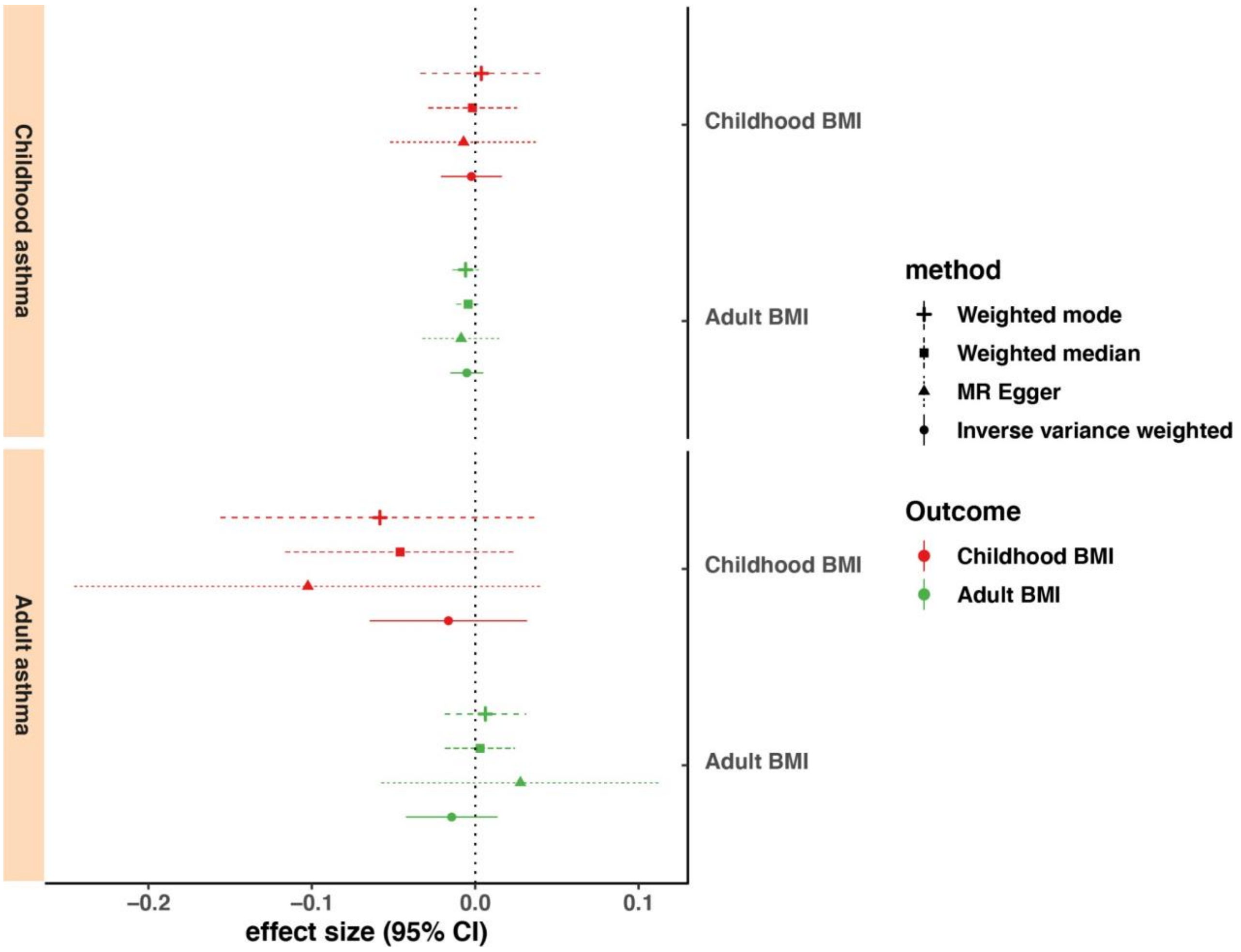

The x-axis is the effect size and 95% confidence interval of the effects for asthma on BMI risk. The vertical dashed line is the reference at =1. Different Mendelian randomization methods are displayed in different line types. For the binary exposures (childhood and adult asthma), MR method results here are only used to evaluate evidence for causality and the consistency of direction of effect. The interpretation of effect sizes for binary exposures needs more caution.

Supplementary Figure 2. Bayesian colocalization analysis identifies shared causal variants between adult BMI and adult asthma

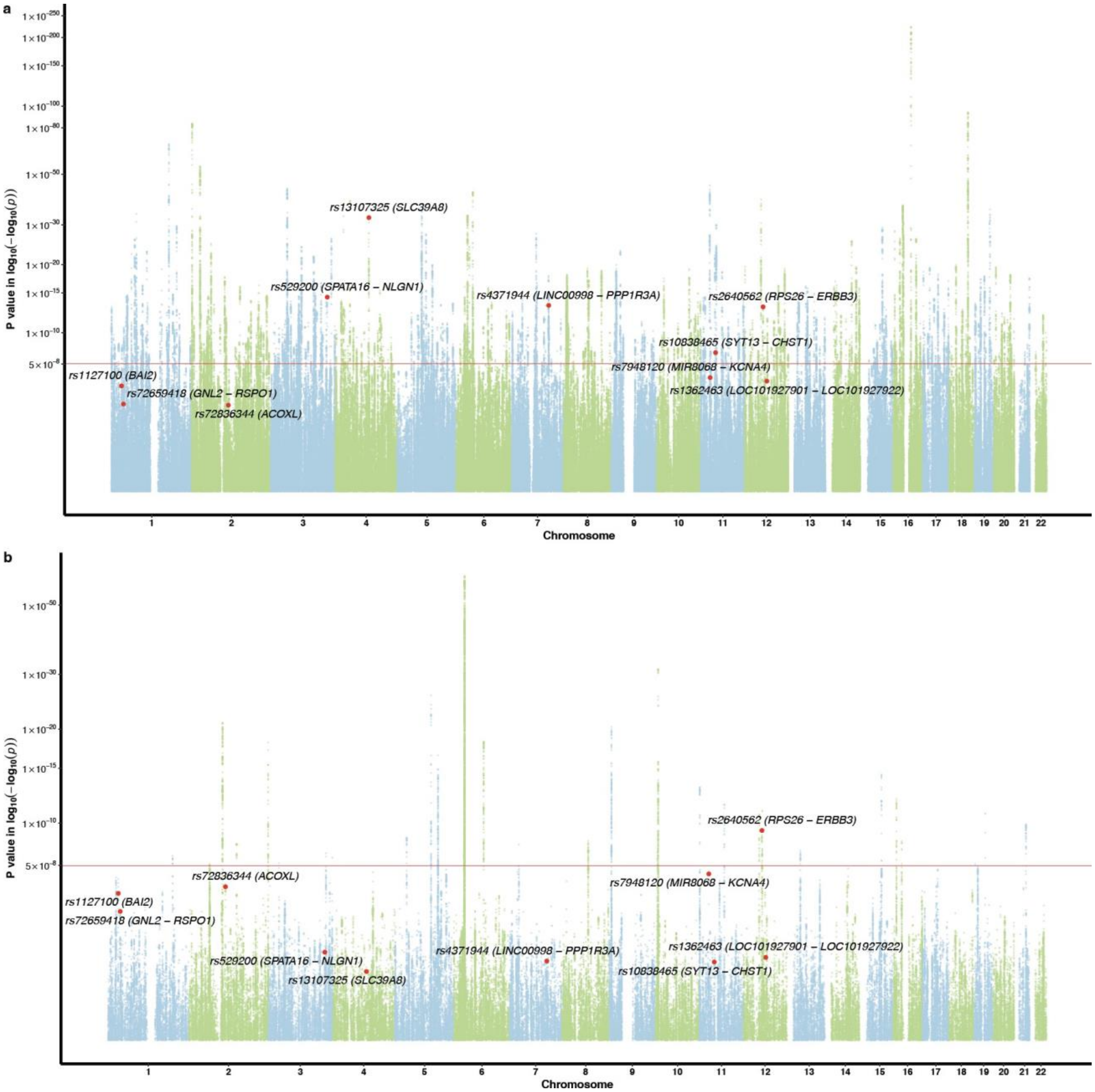

The shared genomic regions are highlighted in red dots and annotated with the best guess causal SNPs and the nearest gene names. The upper panel is the Manhattan plot for adult BMI, and the lower panel is for adult asthma. The red line is the genome-wide significance level (5×10^{-8}). The P values are truncated at 0.01.

Supplementary Figure 3. Association of IL-6 with asthma and wheezing in the Boston Birth Cohort

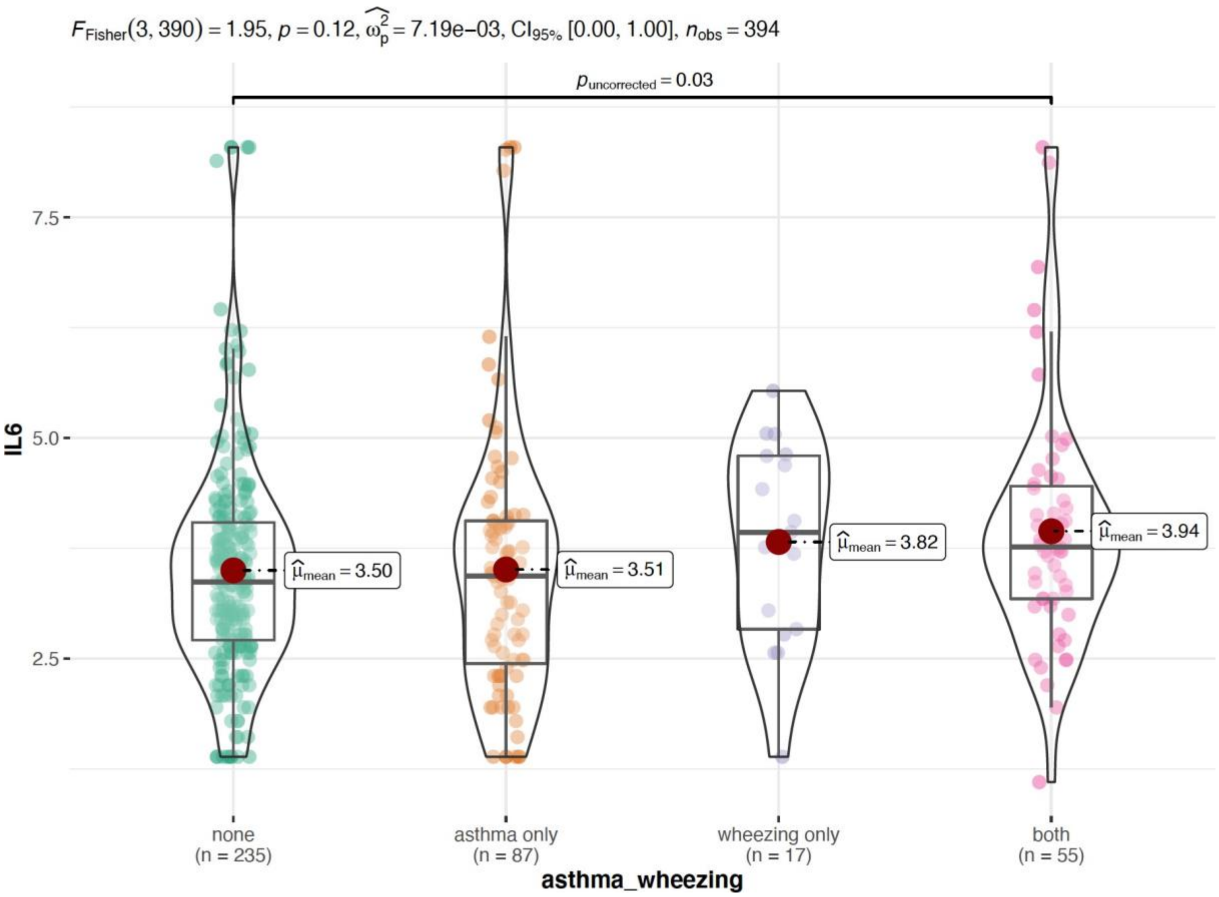

In the BBC, 394 children (2 years and older) with both IL-6 and asthma diagnosis information were included. The participants were separated into four groups: both (asthma+wheezing), asthma only, wheezing only and none (the reference group). For one unit increase of IL6 (after log transformation), the odds ratio of both asthma and wheezing was 1.28 (95% CI: 1.03-1.59, P=0.026) after adjusting for sex, age and enthnic groups. The boxplot shows the center line for median value; box limits for upper and lower quartiles; whiskers for 1.5x interquartile range.

Supplementary Table 1. A summary of previous studies.

Study (PMID)	Exposure: BMI/obesity	Outcome: Asthma	Limitations
Au Yeung et al., 2021 (33051271)[13]	14 BMI-related SNPs derived from **children**	**All asthma** in the UK Biobank	This study did not distinguish adult and childhood asthma. It is known that adult and childhood asthma have different genetic components. The effect of childhood BMI on childhood asthma was unexplored in this study.
Richardson et al., 2022, (35484151)[14]	Childhood and adult body size	**All asthma** (36.7% childhood onset)	This study used categorized body size GWAS to evaluate the association with asthma. However, it did not distinguish adult and childhood asthma.
Chen et al, 2022, (34856028)[15]	Obesity related SNPs were selected from both **children and adults GWAS**	**Childhood** asthma	This study used one sample MR analysis in Asian population, however, the obesity related SNPs were selected from children and adults GWAS.
Granell et al., 2014 (24983943)[16]	32 BMI-related SNPs derived from **adults**	**Childhood** asthma	This early study included 32 BMI SNPs that were derived from adults. It implicitly assumed that adult BMI had the same genetic effect with childhood BMI, however, our study explored their different effect to childhood asthma.
Melén et al., 2013 (23517042)[17]	BMI GWAS in 2,691 asthmatic children	No specific outcome (BMI GWAS in 2,691 asthmatic children)	This study used GWAS analysis to identify *DENND1B* variants that may be associated with BMI in asthmatic children, which have very limited overlap with our current study. The authors reported that the association of the novel BMI loci with asthma was not replicated in the independent data sets.
Lee et al., 2021 (33888571)[18]	BMI whole-genome sequencing studies in ~ 1000 children with asthma and their parents (trios).	No specific outcome.	This study used whole sequencing to identify a novel BMI locus (rs9292139) in LOC102724122, which has limited overlap with our current study. The authors did not observe a significant association between the novel BMI loci and asthma

			affection status.
Zhu et al., 2020 (31669095)[19]	**Adult** BMI in UK biobank	**Childhood** asthma and **adult** asthma	This study only evaluated the genetic correlation between adult BMI and early-onset asthma. The effect of childhood BMI on childhood asthma was not explored.

Note: text bolded for emphasis the exposures and outcomes in children or adults.

Supplementary Table 2. Genome-wide genetic correlation between body mass index and asthma in children and adults.

**Traits**	**Childhood BMI**	**Adult BMI**	0.63 (0.03)<br>(P = 2.46 × 10^{-107})
**Childhood Asthma**	0.10 (0.04)<br>P = 0.02	-0.03 (0.02)<br>P = 0.21	-
**Adult Asthma**	0.10 (0.05)<br>P = 0.03	0.24 (0.03)<br>P =1.70 × 10^{-18}	-
0.64 (0.05)<br>(P = 4.52 ×10^{-43})	-	-	-

This table shows the genome-wide genetic correlations (standard error) and their P values between body mass index (BMI) and asthma in children and adults. The upper-right corner of the table shows the genetic correlation between childhood BMI and adult BMI. The bottom-left corner of the table shows the genetic correlation between childhood asthma and adult asthma.

Supplementary Note 1

Obesity-related biomarker GWAS summary statistics: We obtained GWAS summary statistics for obesity-related biomarkers in two major pathways: insulin/insulin-like growth factor (IGF) axis and chronic low-grade inflammation, including adiponectin, C-reactive protein (CRP), IGF-1, interleukin 6 (IL-6), insulin, leptin, resistin, and tumour necrosis factor receptors (TNFR1, TNFR2).[1]

In general, we conducted genome-wide meta-analysis or obtained GWAS summary statistics for obesity related biomarkers using the Nurses' Health Study (NHS), NHSII, and Health Professionals Follow Up Study (HPFS), and available GWAS summary statistics from GWAS Catalog. Heritable covariates were not included in biomarker GWAS. For the obesity-related biomarker in the NHS, NHSII, and HPFS, GWAS were performed within each genotype platform using the RVTESTS software (version 20190205).[2] We adjusted for age, fasting status, cohort (NHS, NHSII, and HPFS), indicators of subcohort outcome for both the original genetic and biomarker studies, and the first four genetic principal components, the residual phenotypes were inversely normal transformed to obtain score statistics (--inverseNormal and --useResidualAsPhenotype in RVTESTS).[2] The GWAS summary statistics from each genotype platform were meta-analyzed across different genotype platforms based on the inverse variance-weighted method (METAL software).[3] In this study, the GWAS summary statistics for IGF-1 and CRP were from the UK Biobank (UKB), where sample overlap with our BMI and asthma GWAS summary statistics could bias MR estimates. A cross-trait LD-score regression method was used to approximate an arbitrary degree of sample overlap and to correct the bias.[4]

Supplementary Table 3. Sample size and sources of obesity-related biomarker GWAS summary statistics

Biomarker	Sample size	Number of genetic instruments	Data resources and References
Adiponectin	Median (IQR): 14242 (14242 - 40853)	23	NHS/NHSII/HPFS and publicly available GWAS summary statistics[5,6]
C-reactive protein (CRP)	Median (IQR): : 480672 (405281 - 480672)	97	UKB, NHS/NHSII/HPFS, and publicly available GWAS summary statistics[7]
IGF-1	353824	307	GWAS Catalog (UKB)[8]
Interleukin 6 (IL-6)	Median (IQR): 10848 (9901-14242)	9	GWAS Catalog (SCALLOP consortium)[9]
Insulin	Median (IQR): 124123 (118059-125397)	36	GWAS Catalog[10]
Leptin	10835 (10835 - 32769)	4	NHS/NHSII/HPFS, INTERVAL study[11], and publicly available GWAS summary statistics[6,12]
Resistin	18353 (IQR: 15975-21747)	13	GWAS Catalog (SCALLOP consortium)[9]
Tumour necrosis factor receptors (TNFR1)	6104	6	NHS/NHSII/HPFS, INTERVAL study[11], and publicly available GWAS summary statistics[6]
TNFR2	14485	4	NHS/NHSII/HPFS, INTERVAL study[11], and publicly available GWAS summary statistics[6]

Supplementary References

1. Nimptsch, K., Konigorski, S. & Pischon, T. Diagnosis of obesity and use of obesity biomarkers in science and clinical medicine. *Metabolism* **92**, 61–70 (2019).
2. Zhan, X., Hu, Y., Li, B., Abecasis, G. R. & Liu, D. J. RVTESTS: an efficient and comprehensive tool for rare variant association analysis using sequence data. *Bioinformatics* **32**, 1423–1426 (2016).
3. Willer, C. J., Li, Y. & Abecasis, G. R. METAL: fast and efficient meta-analysis of genomewide association scans. *Bioinformatics* **26**, 2190–2191 (2010).
4. Mounier, N. & Kutalik, Z. Correction for sample overlap, winner's curse and weak instrument bias in two-sample Mendelian Randomization. *bioRxiv* 2021.03.26.437168 (2021) doi:10.1101/2021.03.26.437168.
5. Dastani, Z. *et al.* Novel loci for adiponectin levels and their influence on type 2 diabetes and metabolic traits: a multi-ethnic meta-analysis of 45,891 individuals. *PLoS Genet.* **8**, e1002607 (2012).
6. Suhre, K. *et al.* Connecting genetic risk to disease end points through the human blood plasma proteome. *Nat. Commun.* **8**, 1–14 (2017).
7. Kanai, M. *et al.* Genetic analysis of quantitative traits in the Japanese population links cell types to complex human diseases. *Nat. Genet.* **50**, 390–400 (2018).
8. Sinnott-Armstrong, N. *et al.* Genetics of 35 blood and urine biomarkers in the UK Biobank. *Nat. Genet.* **53**, 185–194 (2021).
9. Folkersen, L. *et al.* Genomic and drug target evaluation of 90 cardiovascular proteins in 30,931 individuals. *Nat Metab* **2**, 1135–1148 (2020).
10. Chen, J. *et al.* The trans-ancestral genomic architecture of glycemic traits. *Nat. Genet.* **53**, 840–860 (2021).
11. Sun, B. B. *et al.* Genomic atlas of the human plasma proteome. *Nature* **558**, 73–79 (2018).
12. Kilpeläinen, T. O. *et al.* Genome-wide meta-analysis uncovers novel loci influencing circulating leptin levels. *Nat. Commun.* **7**, 1–14 (2016).
13. Au Yeung, S. L., Li, A. M. & Schooling, C. M. A life course approach to elucidate the role of adiposity in asthma risk: evidence from a Mendelian randomisation study. *J. Epidemiol. Community Health* **75**, 277–281 (2021).
14. Richardson, T. G. *et al.* Childhood body size directly increases type 1 diabetes risk based on a lifecourse Mendelian randomization approach. *Nat. Commun.* **13**, 2337 (2022).
15. Chen, Y.-C., Su, M.-W., Brumpton, B. M. & Lee, Y. L. Investigating obesity-related risk factors for childhood asthma. *Pediatr. Allergy Immunol.* **33**, e13710 (2022).

16. Granell, R. *et al.* Effects of BMI, fat mass, and lean mass on asthma in childhood: a Mendelian randomization study. *PLoS Med.* **11**, e1001669 (2014).
17. Melén, E. *et al.* Genome-wide association study of body mass index in 23 000 individuals with and without asthma. *Clin. Exp. Allergy* **43**, 463–474 (2013).
18. Lee, S. *et al.* Novel recessive locus for body mass index in childhood asthma. *Thorax* **76**, 1227–1230 (2021).
19. Zhu, Z. *et al.* Shared genetic and experimental links between obesity-related traits and asthma subtypes in UK Biobank. *J. Allergy Clin. Immunol.* **145**, 537–549 (2020).
