# Supplementary material for: Obesity-related biomarkers underlie a shared genetic architecture between childhood body mass index and childhood asthma
Source: Commun Biol. 2022 Oct 17;5:1098. doi: 10.1038/s42003-022-04070-9 (PMC9576683; doi:10.1038/s42003-022-04070-9)
Supplement: Supplementary file 2 — Description of Additional Supplementary Files [file 42003_2022_4070_MOESM2_ESM.docx]

**File Name:** Supplementary Data 1

**Description:** Source data underlying figures.
